# Study of Viability, Storage Stability, and Shelf Life of Probiotic Instant Coffee *Lactiplantibacillus plantarum* Subsp. *plantarum* Dad-13 in Vacuum and Nonvacuum Packaging at Different Storage Temperatures

**DOI:** 10.1155/2022/1663772

**Published:** 2022-11-17

**Authors:** Syerina Raihatul Jannah, Endang Sutriswati Rahayu, Rini Yanti, Dian Anggraini Suroto, Rachma Wikandari

**Affiliations:** ^1^Department of Food and Agricultural Product Technology, Faculty of Agricultural Technology, Universitas Gadjah Mada, Yogyakarta 55281, Indonesia; ^2^University Center of Excellence for Research and Application on Integrated Probiotic Industry, Universitas Gadjah Mada, Yogyakarta 55281, Indonesia

## Abstract

Probiotic coffee is an alternative to processed coffee that is preferred and can improve the balance of intestinal microflora so that it has a positive impact on health. Cell viability of probiotics may decrease during storage. Factors that can affect viability during storage are storage temperature, packaging, oxygen, and water activity. This study is aimed at evaluating the viability, storage stability, and shelf life of the probiotic instant coffee *Lactiplantibacillus plantarum* subsp. *plantarum* Dad-13 in vacuum and nonvacuum aluminium foil packaging and different storage temperatures. This study used a complete randomised design with three replicates of treatments. They were packaged using 90 *μ*m thick aluminium foil in a vacuum and nonvacuum and stored at 4°C and 30°C for 50 days and 37°C for 15 days. Based on the literature, a temperature of 4°C can maintain the viability of probiotics for more than one month, the temperature commonly used to store dry products is room temperature (30°C), so longer storage (50 days) is tried. Meanwhile, to accelerate the prediction of quality degradation, extreme temperatures were used based on the literature that the viability of probiotics decreased drastically after being stored at 37°C for 7 days, then tried for longer storage (15 days). The evaluation of product was carried by sensory testing by comparing commercial instant coffee. The product has been tested for cell viability, water activity, and shelf life. The result showed that the colour attribute was significantly different for all formulations. The bitterness of probiotic instant coffee differed significantly from other formulations. The commercial instant coffee was preferred by panellist in terms of colour and bitterness. The aroma, sweetness, and overall attributes of all formulations were not significantly different. The cell viability in vacuum was higher than nonvacuum treatment, and it was higher in 4°C. However, cell viability for all treatments and during storage was still above 10^7^ log CFU/g. Water activity in probiotic instant coffee with vacuum packaging is lower than in nonvacuum and stored at 4°C lower than in other temperatures. However, all treatments were still below 0.60. The shelf life of products reaches two years when they are stored in vacuum packaging at 4°C while a temperature of 30°C reaches 3 months. So, the panellists accepted probiotic instant coffee, vacuum packaging, and low temperature could maintain viability, stability, and longer shelf life.

## 1. Introduction

Coffee is one of the drinks that many people like because of its delicious aroma and taste. Coffee contains beneficial compounds such as caffeine and chlorogenic acid. Caffeine can increase the immune system and chlorogenic acid which acts as an antioxidant [1, 2].

There are various kinds of coffee drink products on the market, such as brewed coffee and instant coffee. Instant coffee is ground coffee with a low water content made by spray drying so that it is easily soluble in water [3]. Recently, there has been much demand from the market to increase the functional value of instant coffee products, one of which is the addition of probiotics that can potentially improve health.

Probiotics are live microorganisms that, when consumed in sufficient quantities, could provide a health effect on the host [4]. Probiotics provide health benefits, especially the maintenance of intestinal microflora, protect digestive tract pathogens, increase nutrient utilisation, increase nutritional value of food, lower serum cholesterol, lower blood pressure, anticarcinogenic activity, antioxidant activity, and boost the immune system [5, 6]. The most commonly used probiotic microorganisms are lactic acid bacteria. Generally, lactic acid bacteria are used as probiotics from the *Lactobacillus* group. *Lactiplantibacillus plantarum* subsp. *plantarum* Dad-13 was isolated from dadih and has been tested for safety through rat trials so that consuming it does not negatively impact on the health of mice in general [7, 8]. In addition, the faeces of rats that were given *Lactiplantibacillus plantarum* subsp. *plantarum* Dad-13 increased significantly. From the experiments that have been carried out, this strain was a potential probiotic candidate.

The addition of probiotics to food products must contain probiotics that reach more than 10^6^ CFU/g [9]. Cell viability of probiotics in instant coffee products must be more than 10^6^ CFU/g so that it can have a positive effect on health. Adding probiotics to food products is a challenge that can trigger a decrease in probiotic cells during the product manufacturing process. Therefore, to maintain the viability of probiotics in instant coffee products, it is necessary to add the probiotic powder to the dry mix of instant coffee powder (dry mix).

Commercial instant coffee was used as a control for comparison with probiotic instant coffee in product preference testing. Furthermore, this study tested the viability of probiotic instant coffee, water activity, and shelf life [10, 11]. Cell viability of probiotics may decrease during storage. The decrease in viability is influenced by storage temperature, O₂, and water activity of the material. There was a decrease in cell viability in *Lactobacillus paracasei* SNP2 if the incubation temperature is above 30-45°C [10]. O₂ in packaging can increase product shelf life. In addition, aw can affect the viability of probiotics during storage. If the aw in the sample increases, the probiotic viability decreases. Therefore, to maintain high viability, it is essential to keep the powder in suitable packaging materials and storage conditions to protect the dry cells from exposure to oxygen, moisture, and light. It is found that in *Lactobacillus plantarum* Dad-13 spray-dried starter cultures in vacuum polyethylene packaging, the number of viable cells was maintained at 10^9^ CFU/g even after storage at 4°C for 4 weeks [12].

The models for shelf-life estimation products can be conducted in various ways, such as direct and indirect methods. The indirect method is testing with Accelerated Shelf-Life Testing (ASLT) or predictive models. ASLT is an approach to determining the shelf life of food products by storing the product in an environment that could accelerate the decline in critical parameters such as temperature and humidity [13]. Shelf-life testing of probiotic instant coffee uses probiotic cell viability as a critical parameter. This study has evaluated the viability, storage stability, and shelf life of the probiotic instant coffee *Lactiplantibacillus plantarum* subsp. *plantarum* Dad-13 in vacuum and nonvacuum aluminium foil packaging and different storage temperatures.

## 2. Materials and Methods

The material used in making probiotic instant coffee was robusta coffee powder obtained from the production of Merapi Coffee, Yogyakarta, Indonesia, with the specification of the coffee was roasted using an outlet temperature of 200°C for 90 min (well done), powder size 80 mesh; probiotic bacteria (*Lactiplantibacillus plantarum* subsp. *plantarum* Dad-13) isolated from the dadih by the Food and Nutrition Culture Center Collection (FNCC), Center for Food and Nutrition Studies, Universitas Gadjah Mada, Yogyakarta, Indonesia; food grade maltodextrin (P.T. Garuda Mas Lestari, Bandung, Indonesia); sugar (P.T. Sugar Group Companies (S.G.C.), Lampung, Indonesia); mineral water brand Aqua (P.T. Tirta Investama, Jakarta, Indonesia); and another reagent used in this study was the analytical grade.

### 2.1. Preparation of Instant Coffee

The instant coffee was prepared according to [14] with minor modifications. The ground roasted coffee was extracted with boiling water (1 : 10 *w*/*w*), stirred for 20 min, and filtered. The coffee solution was added with 20 g/100 mL maltodextrin. Maltodextrin was added gradually to the solution using an Ultra-Turrax T-50 basic homogenizer (IKA-Werke, Staufen, Germany) at 4000 rpm for 5 min. The drying process was carried out using a mini spray dryer B-290 (BUCHI Labortechnik AG, Flawil, Switzerland). The inlet and outlet temperatures were 120°C and 64-83°C, respectively. After cooling to room temperature, the coffee samples were packaged.

### 2.2. Preparation of Probiotic Instant Coffee

Instant coffee was made from a mixture of instant coffee with probiotic *Lactobacillus* Dad-13 in a formulation of 20 g containing 10^9^ CFU/g of probiotics. *Lactiplantibacillus plantarum* subsp. *plantarum* Dad-13 dry biomass is mixed with probiotic instant coffee and stirred until well blended according to the following calculation

(1) $$\begin{array}{c} \frac{\text{ gram initial probiotic}\times \text{initial probiotic }\mathrm{rank}}{\text{total ingredients}}=\mathrm{rank}\text{ product}. \end{array}$$

One gram of the sample was then stored in an aluminium foil package with a thickness of 90 *μ*m and measuring 2 × 3 cm, which was vacuumed using a 200A vacuum packer (Henkelman, Ashford, UK) and nonvacuum sealed using an impulse sealer matrix PFS-400 (SAP, Malaysia). After packaging, the samples were stored following the storage temperature.

### 2.3. Sensory Evaluation

Sensory test of probiotic instant coffee drink based on preference test was determined according to [15] with minor modifications. This drink uses 20 g of probiotic instant coffee (17 : 3 g/g) and 8 g of sugar brewed in 150 mL cold mineral water. Probiotic instant coffee has approved a permit with protocol number KE/FK/0467/EC/2022 from the Medical and Health Research Ethics Committee (MHREC), Faculty of Medicine, Public Health and Nursing, Universitas Gadjah Mada-Dr. Sardjito General Hospital, Yogyakarta, Indonesia. The coffee was served to 68 panellists from students and staff of Universitas Gadjah Mada, Yogyakarta. Determination of the number of panellists based on the Raosoft website (http://www.raosoft.com/samplesize.html) with the sample population used coming from the total population of D. I Yogyakarta, Indonesia, as many as 3,677,446 people in 2021 (Bureau of Governance for the Regional Secretariat of D. I Yogyakarta, Indonesia). Panellists were asked to rate the sample based on their preferences according to the provided rating scale. The quality attributes assessed by the panellists were colour, aroma, sweetness, bitterness, aftertaste, and overall rating. Before the evaluation, the panellists were told that the test score scale used was between 1 and 7; a value of 1 means that the panellists do not like it very much, and a value of 7 means that the panellists really like it. The product intensity profile uses a scale of 1 to 7 with descriptions of colour 1: very light and 7: very dark; aroma 1: very absent and 7: very high; sweetness 1: very not sweet and 7: very sweet; and bitter taste 1: not very bitter and 7: very bitter.

### 2.4. Probiotic Cell Viability *Lactiplantibacillus plantarum* Subsp. *plantarum* Dad-13 during Storage

Cell viability was performed based on [12] with minor modifications. Cell viability in probiotic instant coffee used the total plate count method with 10 days at 4°C and 30°C, while the storage interval was three days at 37°C. Probiotic instant coffee 1 g was dissolved in 9 mL 0.85% NaCl (10^1^ dilution). Then 1 mL was taken and 9 mL of 0.85% NaCl was added for a 10^2^ dilution until the desired dilution. The last three dilutions were taken 1 mL to be inoculated in MRS agar with petri dishes. Then, the media was incubated for 48 h at 37°C. The number of bacteria was counted with the Quebec Colony Counter. Colonies formed were counted and expressed as log CFU/g. The formula used to calculate the number of colonies according to [16], where S.I. is the number of colonies; ∑C is the number of all colonies on all plates; *n*_1_ is the number of petri dishes in the first dilution; *n*_2_ is the number of petri dishes in the second dilution; and *d* is the dilution at which the first count is obtained (first dilution).

(2) $$\begin{array}{c} \text{S}.\text{I}.=\frac{\left(\Sigma C\right)}{\left(1\times n_{1}\right)+\left(0.1\times n_{2}\right)}xd. \end{array}$$

Colonies that could be included in the calculation are those numbering from 25 to 250 cells [16]. If the number of bacteria is less than 25, it is called EAPC (Estimated Aerobic Plate Count) and more than 250 is called TNTC (Too Many To Count).

### 2.5. Water Activity

Measurements of aw in the probiotic instant coffee were determined using an Aqualab PAWKIT (Decagon Devices, Inc., Pullman, WA, USA).

### 2.6. Estimating the Shelf Life of Probiotic Instant Coffee with ASLT Method

Estimating shelf life used the Accelerated Shelf-Life Testing (ASLT) method [13]. Probiotic instant coffee was packaged in aluminium foil with a thickness of 90 *μ*m and size of 2 × 3 cm, vacuum and nonvacuum stored at 4°C, 30°C, and 37°C. The critical parameter is the cell viability of probiotic bacteria. Viability measurements were done every 10 days for 50 days storage on samples kept at 4°C and 30°C, while samples kept at 37°C were measured for viability every 3 days for 15 days. The results of viability decrease form the samples can follow reactions of order 0 and order 1. A graph showing relationship between the value of *k* (quality change rate) and *t* (storage time) was made to determine the order of 0 and 1. The regression equation was selected based on the order with a higher coefficient of determination. Each equation has an intercept value (a) as the viability of probiotic cells on day 0 of storage and a slope value (b) as a reaction rate constant for decreasing the quality of probiotic instant coffee products, which is described by a decrease in probiotic cell viability (*k*_0_). The dependence of the Arrhenius constant (*k*) on temperature (*T*) is explained by the following Arrhenius equation:

(3) $$\begin{array}{c} \text{Ln k}=\ln\;k_{0}–\frac{Ea}{RT}. \end{array}$$

The following reaction kinetics calculated the shelf-life determination, where *N*_0_ or ln *N*_0_ is the number of probiotic cells on day 0; *N*_*t*_ or ln *N*_*t*_ is a critical limit of total probiotic cells.

(4) $$\begin{array}{c} \text{Order }0:\;\text{Ts}=\frac{\left(N_{0-}N_{t}\right)}{k},\\ \text{Order }1:\;\text{Ts}=\frac{\left(\;\ln\;N_{0-}\ln\;N_{t}\right)}{k}. \end{array}$$

### 2.7. Statistical Analysis

The experiment was repeated in triplicate. Sensory tests using spider web, viability using Microsoft excel, and water activity were reported as mean ± standard deviation. All the statistical analysis was performed using the SPSS Statistics 26 software (IBM Corporation, New York, USA). The determination of statistical significance level (*p* < 0.05) using one-way ANOVA with Duncan post hoc test was conducted.

## 3. Results and Discussion

### 3.1. Sensory Evaluation

**Figure 1:**
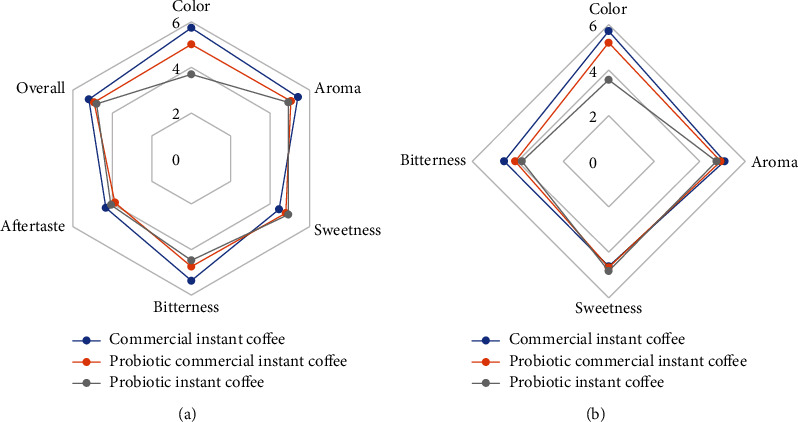
(a) Hedonic sensory attributes and (b) sensory intensity attributes. The test values used are between 1 and 7, i.e., 1 (extremely dislike), 2 (dislike very much), 3 (dislike slightly), 4 (neither like nor dislike), 5 (like slightly), 6 (like very much), and 7 (extremely like).

The results of the sensory evaluation of probiotic instant coffee based on panellist preferences are shown in Figure 1 (hedonic test and intensity test). This is because the formulation of commercial instant coffee as a control was not added with probiotics so that it did not change its original colour (dark); while in both formulations, probiotics were added with the same weight. However, the formulation of probiotic instant coffee has a different colour (brighter) from the two formulations before the administration of probiotics, so the colour became lighter after the administration of probiotics. The probiotics' addition can change the colour due to the manufacture of probiotic powder using a carrier material, skim milk. So, the panellists prefer the formulation of commercial instant coffee colour attribute of 5.74 (like slightly). In contrast, the lowest formulation of probiotic instant coffee is 3.69 (dislike slightly).

Taste is the most critical quality parameter for coffee products [17]. Hedonic testing and sweetness intensity did not significantly differ between the three formulations. The reason is that the use of sugar in this test has the same weight. However, the formulation of probiotic instant coffee is the most preferred 4.93 (like slightly). Commercial instant coffee had the highest hedonic and bitterness intensity 5.37 (like slightly) and 4.59 (neutral) and was significantly different from the two formulations (Figures 1(a) and 1(b)).

In contrast, the formulations of probiotic commercial instant coffee and probiotic instant coffee were not significantly different. The reason is that adding sweetness to coffee can reduce the bitter taste. In the hedonic aftertaste attribute, the highest formulation was commercial instant coffee 4.34 (neither like nor dislike), while the lowest was formulation probiotic commercial instant coffee 3.87 (dislike slightly). However, the three formulations of the aftertaste attribute had no significant difference. So, the panellists can accept the preference of probiotic instant coffee on taste (sweet taste, bitter taste, and aftertaste). For overall acceptability, there was no significant difference between the formulation of probiotic instant coffee and the other two formulations, so the addition of probiotics does not affect the sensory attributes.

### 3.2. Probiotic Cell Viability *Lactiplantibacillus plantarum* Subsp. *plantarum* Dad-13 during Storage

**Figure 2:**
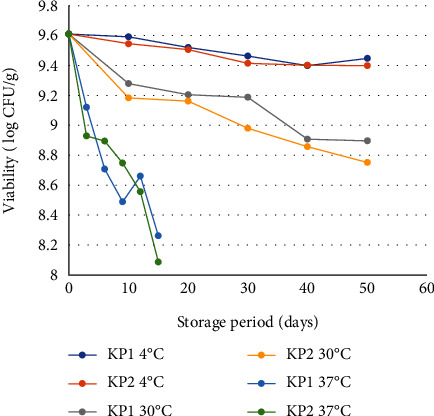
Probiotic instant coffee viability after storage. KP1: vacuum-packaged probiotic instant coffee and KP2: probiotic instant coffee nonvacuum packaging.

A temperature of 37°C can cause a significant loss of cell viability [18]. In addition, oxygen and moisture content may be detrimental to dry cultures. Observation of cell viability of vacuum-packed and nonvacuum probiotic instant coffee at different storage temperatures from the initial day of storage is presented in Figure 2. It was found that vacuum-packed probiotic instant coffee had higher cell viability than that of nonvacuum samples. Vacuum-packaged probiotic instant coffee stored at 4°C achieved the highest viability (9.45 log CFU/g) among all treatments stored at 30°C and 37°C. The reason might be because cells are stored at temperatures close to 0°C, and they can reduce the rate of adverse chemical reactions [19]. In addition, the presence of O₂ in the packaging causes oxidation of membrane lipid and protein denaturation resulting in degradation of macromolecular in bacterial cells [18].

Samples KP1 at 37°C increased by 0.17 log CFU/g from days 9 to 12. Days 6 and 9 were lower than KP2, possibly due to the use of sealers when the vacuum was not tight or due to the mechanical damage to the packaging resulted in air exchange in the sample, thus the sample is damaged faster. While the KP2 packaging used is in good condition so that there is no air exchange and is only inside the package. Then on the 12 days and 15 days, the KP1 packaging used was in good condition so the sample condition was better than that of KP2. Therefore, there is a line crossing between KP1 to KP2. Meanwhile, the lowest viability (8.75 log CFU/g) was in packaged probiotic instant coffee nonvacuum which was stored at 37°C. In line with research by [20], the viability of probiotic *Lactobacillus paracasei* NFBC 338 decreased significantly after seven days of storage at 37°C. However, the viability of probiotic cells with vacuum and nonvacuum packaging at all storage temperatures was still more than 6 log CFU/g after 50 days of storage (4°C and 30°C) and 15 days of storage (37°C).

### 3.3. Water Activity

**Figure 3:**
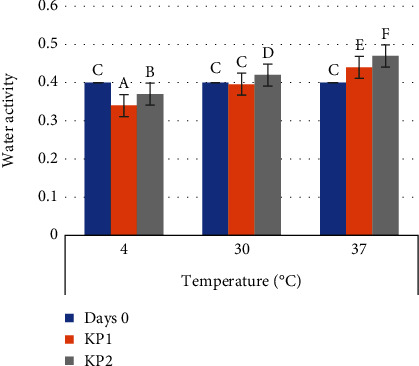
Water activity after storage. KP1: vacuum-packaged probiotic instant coffee and KP2: probiotic instant coffee nonvacuum packaging. Values are presented as mean ± SD, *n* = 6. Values with the same letter are not significantly different (*p* < 0.05).

Powder products having with water activity lower than 0.6 are stable against microbial contamination and lipid oxidation [9, 21]. The value of water activity of probiotic vacuum instant coffee and nonvacuum at different storage temperatures is presented in Figure 3.

The result obtained that the water activity in vacuum packaging for all storage temperatures was significantly different from that of nonvacuum packaging. Water activity in vacuum-packaged samples is lower than that of nonvacuum packaging. It is in line with the research [22] that using vacuum packaging on guava powder can inhibit the increase in water activity. Storage temperature also affects water activity. The higher the storage temperature, the greater the water activity of the material. The activity of probiotic instant coffee water was significantly different for all storage temperatures. The lowest water activity was 0.34 on vacuum-packed samples at 4°C storage temperature. In line with the research that the water activity of the spray dry yoghurt starter culture on vacuum-packaged polyethylene stored at 4°C was 4.09 to 4.24% [12]. Water activity is also related to relative humidity. Changes in relative humidity at a specific temperature can cause fluctuations (increase or decrease) in water content. Therefore, after storage, there was a decrease in product water activity [23]. While the highest water activity of 0.47 was obtained from nonvacuum packaging samples at a storage temperature of 37°C. As in the research [22], increasing temperature and storage time can increase water activity. However, the activity of water with vacuum and nonvacuum packaging is still below 0.6, indicating the stability of the powder against harmful chemical and microbiological reactions.

### 3.4. Estimating the Shelf Life of Probiotic Instant Coffee with the ASLT Method

The observation of shelf life's results based on the viability of probiotics that has been carried out on probiotic instant coffee using vacuum and nonvacuum packaging with three different storage temperatures of 4°C, 30°C for 50 days of storage, and 37°C for 15 days of storage can be seen in Table 1. Based on the results of plotting data between the decrease in cell viability of instant coffee probiotics and storage time on order 0 and order 1, the value of the coefficient of determination (*R*^2^) in the data plotted with order 1 is higher than order 0, so the regression equation on order 1 is chosen as a calculation of shelf life. The estimated shelf life of probiotic instant coffee shows that probiotic instant coffee stored at 4°C has a much longer shelf life than products stored at 30°C and 37°C. Vacuum-packaged probiotic instant coffee stored at 4°C had the most extended shelf life of 726 days (2 years). In line with the research results that at a temperature of 4°C-7°C, the viability of probiotic cells can survive [12]. Furthermore, studies have shown that the viability of encapsulated probiotics stored at 4°C was higher than that of cells stored at 25°C [9]; whereas at the temperature of 30°C storage in vacuum packaging, the shelf life is longer than in nonvacuum packaging. Vacuum-packaged products with a storage temperature of 30°C have a shelf life of 95 days (3 months). Based on the data obtained, probiotic instant coffee per serving (20 g) contains 2 × 10^8^ CFU/g.

## 4. Conclusions

Currently, functional foods containing probiotics are attracting attention in the market. This study makes a functional product from probiotic instant coffee by determining its shelf life. The product was packaged in aluminium foil packaging vacuum and nonvacuum, which was stored at 4°C, 30°C, and 37°C to find out if the packaging used could maintain viability and the shelf life of the sample. Based on the hedonic test and sensory intensity, the probiotic instant coffee formulation was acceptable to the panellists. Moreover, vacuum packaging can affect water viability and activity. The viability of probiotic cells was vacuum packed with aluminium foil and stored at a higher temperature than nonvacuum packaging. This proves that aluminium foil vacuum packaging can protect probiotic instant coffee grounds against oxygen, moisture, and light that can damage cells. Probiotic instant coffee stored in cold conditions has shown higher viability and longer shelf life than samples stored at room temperature.

## Figures and Tables

**Table 1:** Shelf life of probiotic instant coffee.

Temperature (°C)	Treatment	*k*	ln no.	ln Nt	ts	
4	KP1	0.008273	25.12264	19.11383	726.2952	2 years, 6 days
	KP2	0.009139	25.12264	14.50866	657.4686	1 year, 8 months
30	KP1	0.063087	25.12264	19.11383	95.24586	3 months, 5 days
	KP2	0.069778	25.12264	14.50866	86.11322	2 months, 26 days
37	KP1	0.102847	25.12264	19.11383	58.42497	1 month, 28 days
	KP2	0.113788	25.12264	14.50866	52.80715	1 month, 22 days

KP1: vacuum-packaged probiotic instant coffee, KP2: probiotic instant coffee nonvacuum packaging, k: 1/T (storage temperature), ln no.: the number of probiotic cells on day 0, ln Nt: a critical limit of total probiotic cells, and ts: shelf life (days).

## Data Availability

The raw data used to support the findings of this study have been deposited in the Universitas Gadjah Mada repository **(**https://repository.ugm.ac.id/277719/**).** Raw data is also available upon request to the corresponding author.
